# Mosquito-Disseminated Insecticide for Citywide Vector Control and Its Potential to Block Arbovirus Epidemics: Entomological Observations and Modeling Results from Amazonian Brazil

**DOI:** 10.1371/journal.pmed.1002213

**Published:** 2017-01-17

**Authors:** Fernando Abad-Franch, Elvira Zamora-Perea, Sérgio L. B. Luz

**Affiliations:** 1 Laboratório de Triatomíneos e Epidemiologia da Doença de Chagas, Centro de Pesquisa René Rachou, Fundação Oswaldo Cruz, Belo Horizonte, Minas Gerais, Brazil; 2 Laboratório de Ecologia de Doenças Transmissíveis na Amazônia, Instituto Leônidas e Maria Deane, Fundação Oswaldo Cruz, Manaus, Amazonas, Brazil; Mahidol-Oxford Tropical Medicine Research Unit, THAILAND

## Abstract

**Background:**

Mosquito-borne viruses threaten public health worldwide. When the ratio of competent vectors to susceptible humans is low enough, the virus’s basic reproductive number (*R*_0_) falls below 1.0 (each case generating, on average, <1.0 additional case) and the infection fades out from the population. Conventional mosquito control tactics, however, seldom yield *R*_0_ < 1.0. A promising alternative uses mosquitoes to disseminate a potent growth-regulator larvicide, pyriproxyfen (PPF), to aquatic larval habitats; this kills most mosquito juveniles and substantially reduces adult mosquito emergence. We tested mosquito-disseminated PPF in Manacapuru, a 60,000-inhabitant city (~650 ha) in Amazonian Brazil.

**Methods and Findings:**

We sampled juvenile mosquitoes monthly in 100 dwellings over four periods in February 2014–January 2016: 12 baseline months, 5 mo of citywide PPF dissemination, 3 mo of focal PPF dissemination around *Aedes*-infested dwellings, and 3 mo after dissemination ended. We caught 19,434 juvenile mosquitoes (66% *Aedes albopictus*, 28% *Ae*. *aegypti*) in 8,271 trap-months. Using generalized linear mixed models, we estimated intervention effects on juvenile catch and adult emergence while adjusting for dwelling-level clustering, unequal sampling effort, and weather-related confounders. Following PPF dissemination, *Aedes* juvenile catch decreased by 79%–92% and juvenile mortality increased from 2%–7% to 80%–90%. Mean adult *Aedes* emergence fell from 1,077 per month (range 653–1,635) at baseline to 50.4 per month during PPF dissemination (range 2–117). Female *Aedes* emergence dropped by 96%–98%, such that the number of females emerging per person decreased to 0.06 females per person-month (range 0.002–0.129). Deterministic models predict, under plausible biological-epidemiological scenarios, that the *R*_0_ of typical *Aedes*-borne viruses would fall from 3–45 at baseline to 0.004–0.06 during PPF dissemination. The main limitations of our study were that it was a before–after trial lacking truly independent replicates and that we did not measure mosquito-borne virus transmission empirically.

**Conclusions:**

Mosquito-disseminated PPF has potential to block mosquito-borne virus transmission citywide, even under adverse scenarios. Our results signal new avenues for mosquito-borne disease prevention, likely including the effective control of *Aedes*-borne dengue, Zika, and chikungunya epidemics. Cluster-randomized controlled trials will help determine whether mosquito-disseminated PPF can, as our findings suggest, develop into a major tool for improving global public health.

## Abstract 2

Fernando Abad-Franch and colleagues report entomological observations before and after citywide mosquito control in Amazonian Brazil, and model the estimated impact on transmission of mosquito-borne viruses.

## Author Summary

**Why Was This Study Done?:**

- Urban mosquitoes are global public health threats. They transmit dengue, Zika, and many other diseases for which vaccines or drugs are not available.
- Mosquito control is the key to preventing these diseases, yet conventional control tactics seldom yield satisfactory results. One key drawback is that many mosquitoes (especially *Aedes*) use small, hidden, or inaccessible breeding sites (aquatic larval habitats) that often remain untreated during control campaigns.
- One way of increasing the fraction of breeding sites that are treated is to use the mosquitoes themselves to transfer pyriproxyfen (PPF), a potent juvenile-killing insecticide, from resting sites to untreated breeding sites; there, PPF impedes juvenile mosquito development.

**What Did the Researchers Do and Find?:**

- Working with municipal vector control staff, we tested mosquito-disseminated PPF in a 60,000-inhabitant town of central Amazonia, in Brazil. We sampled juvenile mosquitoes monthly in 100 dwellings over 12 baseline months, eight months of PPF dissemination (five months citywide and three months focal), and three months post-dissemination.
- We caught 12,817 *Aedes albopictus* and 5,346 *Ae*. *aegypti* juveniles, and kept them in the laboratory to measure juvenile mortality and adult emergence.
- Following PPF dissemination, we observed an 80%–90% decrease in *Aedes* juvenile catch, while *Aedes* juvenile mortality increased from 2%–7% to 80%–90%. Adult *Aedes* emergence dropped by 96%–98%, such that the number of females emerging per person decreased to 0.002–0.129 females per person-month.
- Mathematical models predict that this reduction would effectively block transmission of mosquito-borne viruses like dengue, Zika, or chikungunya.

**What Do These Findings Mean?:**

- Our findings suggest that mosquito-disseminated PPF has the potential to become an important public health tool; larger, carefully designed trials are now necessary to determine the impact of this tactic on disease transmission.

## Introduction

Fast global spread of mosquito-borne viruses is among the most pressing contemporary public health challenges [1,2]. The dengue, West Nile, and Japanese encephalitis viruses are well-known mosquito-transmitted pathogens, but we are currently witnessing the emergence of novel threats including chikungunya and Zika [1–6]. Both African in origin, these two viruses are causing large epidemics in the Americas and more restricted outbreaks in Europe, Southeast Asia, and the Pacific [5–7]. Ongoing Zika epidemics are particularly worrying because infection with this virus can cause Guillain-Barré syndrome and congenital central nervous system malformations including microcephaly [6–13].

*Aedes aegypti* and *Ae*. *albopictus* are considered the main urban vectors of dengue, Zika, and chikungunya, while *Culex* spp. mosquitoes transmit West Nile and Japanese encephalitis viruses [1–7]. The presence of urban mosquito vectors also increases the emergence or re-emergence potential of other viruses including yellow fever and Mayaro [1,2]. Effective vaccines exist for yellow fever and Japanese encephalitis, and recent advances in dengue [14] and Zika [15] vaccine development are relatively encouraging. However, major challenges remain (e.g., [16,17]), and for most mosquito-borne viral infections vector control is still the cornerstone of disease prevention [3,18,19]. In theory, effective control of disease spread requires lowering the ratio of competent vectors to susceptible human hosts below a critical threshold value, which, in turn, brings an infection’s basic reproductive number, *R*_0_, below unity [20–22]. *R*_0_ is a fundamental quantity in infectious disease epidemiology [22]. It measures the number of new (secondary) cases that arise from a primary (index) case entering a susceptible host population; with *R*_0_ < 1.0, each case produces, on average, less than one new infection, and the disease fades out from the host population. *R*_0_, then, provides also a measure of the control effort needed to effectively stop transmission [20–22].

Despite the large (and mounting) burden imposed by *Aedes*- and *Culex*-transmitted viruses [1,2,4–13,23,24], current mosquito control tactics have often failed to reliably reduce vector:human ratios to values that would keep *R*_0_ below the 1.0 threshold [18,19,25]. Mosquito control tactics usually combine insecticide spraying to kill adult mosquitoes with the identification and elimination of mosquito breeding sites (i.e., aquatic larval habitats) to limit juvenile mosquito numbers [3,18,19,25]. Unfortunately, insecticide spraying has only transient effects on the adult mosquito population, and the proportion of breeding sites that are detected and treated or eliminated (“breeding-site coverage”) is often so low as to render control campaigns largely ineffective (see [18,25]).

An attractive way to increase breeding-site coverage is to use adult mosquitoes to disseminate tiny particles of juvenile-killing insecticides (larvicides or pupicides) to breeding sites [26,27]. One such insecticide is pyriproxyfen (PPF), an insect juvenile hormone analogue that kills mosquito juveniles at minute doses and can safely be used even in drinking water [3,27,28]. Mosquito-disseminated PPF has been shown to yield high breeding-site coverage and large reductions of adult mosquito emergence across a tropical neighborhood [29]. One crucial open question is whether mosquito-disseminated PPF can effectively reduce mosquito populations at the spatial scale relevant for vector control and disease prevention—the scale of cities and towns. To address this question, we conducted a 2-y trial in a Brazilian Amazon city. First, we asked whether and to what extent some key demographic parameters of local mosquito populations (with a focus on *Ae*. *aegypti* and *Ae*. *albopictus*) would change following citywide deployment of mosquito-disseminated PPF. We then used simple deterministic models to explore the possible impact of observed changes in female *Aedes* emergence on the basic reproductive number, *R*_0_, for dengue and similar pathogens, including Zika, under epidemiological-entomological scenarios ranging from somewhat optimistic to essentially catastrophic.

## Methods

This project was led by the Fundação Oswaldo Cruz (Brazilian Ministry of Health) in a joint initiative with local state and municipal health departments. Formal approval was not required for urban mosquito collection.

### Setting and Mosquito Surveillance

**Fig 1:**
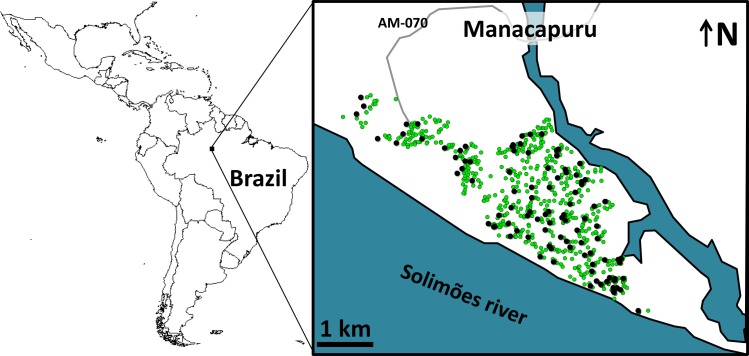
Study site: the city of Manacapuru, state of Amazonas, Brazil. The black circles indicate the location of the 100 dwellings monitored for mosquito vectors; the green circles indicate the location of the 1,000 pyriproxyfen dissemination stations. Circles are overlaid on a schematic of Manacapuru; locations are approximate. The map of Latin America was drawn using the maps library in R 3.1.2 (https://www.r-project.org/).

We conducted a 2-y trial in Manacapuru, a 60,000-inhabitant city (~13,500 dwellings in ~650 ha) in the Brazilian Amazon (data from the Brazilian Institute of Geography and Statistics; http://www.ibge.gov.br/) (Fig 1). We selected 100 dwellings roughly evenly distributed across the city (Fig 1) for mosquito surveillance including two surveys per month from February 2014 to January 2016 (except that no surveys were conducted in November 2015, and just one survey in February 2015 and January 2016; see S1 Data). Residents in these 100 dwellings gave written informed consent to participate in the study.

Each month, we set four sentinel breeding sites (SBSs; two per survey) in each surveillance dwelling. SBSs were 580-ml dark-brown plastic cups containing 400 ml of tap water; they were retrieved after 5–6 d of operation, and their contents kept in the laboratory as previously described [29]. We then recorded (a) the number of juvenile mosquitoes developing or dying in each SBS and (b) the number of adult mosquitoes emerging from each SBS. These data allowed us to assess monthly, for four mosquito taxa (*Aedes aegypti*, *Ae*. *albopictus*, *Culex* spp., and *Limatus* spp.), the following metrics: (a) house infestation, measured as the percent of surveillance dwellings with at least one juvenile mosquito; (b) juvenile mosquito catch, or the number of larvae in each SBS; (c) juvenile mosquito mortality, or the proportion of mosquito juveniles that died before reaching the adult stage in each SBS; and (d) adult mosquito emergence, or the number of adults emerging from each SBS. Here, we focus on our results on juvenile mosquito catch and adult mosquito emergence—and particularly on the epidemiologically most relevant quantity, female mosquito emergence. Full raw data are provided in S1 Data.

### Intervention

In March 2015, after 1 y of monthly monitoring, the intervention started. Citywide PPF dissemination occurred from March through July 2015. Working under our supervision, municipal vector control staff deployed 1,000 PPF dissemination stations (DSs) scattered over the entire urban area (Fig 1); all site owners gave oral informed consent. DSs were 2-l plastic cups containing 600–700 ml of tap water and with the inner wall lined with black, Oxford-type polyester cloth dusted with 5 g of PPF 0.5% (SumiLarv 0.5G; Sumitomo Chemical, Tokyo) ground to fine powder (see also [29]). Municipal vector control staff visited DSs fortnightly for maintenance (re-dusting with PPF and refilling with water). Logistic constraints, however, precluded DS maintenance in some city sectors at some time points (see S1 Data and S1 Fig); we investigated the possible effects of these operational failures using generalized linear mixed models (GLMMs) (see below).

From August through October 2015, PPF dissemination was scheduled to be “focal”—i.e., limited to dwellings with evidence of infestation by *Aedes* spp. based on the SBS surveillance. Again, logistic constraints did not allow for full coverage, with focal dissemination not taking place in eight of the 37 dwellings found to be infested at least once over this 3-mo period; in addition, our field team noted that PPF used in focal dissemination in October 2015 (26 dwellings) was not ground to sufficiently fine powder (see S1 Data and S1 Fig). Final PPF dissemination occurred in October 2015, with SBS-based monitoring maintained until January 2016. Thus, the trial spanned 12 mo before PPF dissemination, 5 mo of citywide PPF dissemination, 3 mo of focal PPF dissemination, and 3 mo after PPF dissemination stopped. Importantly, conventional *Aedes* control measures (active breeding-site searches and breeding-site elimination by municipal vector control staff) were in place over the first 12 and last 6 mo of the trial—i.e., over the periods with no citywide PPF dissemination.

### Descriptive Analyses

We first described our data using graphs and tables, and calculated summary statistics including percentages with score 95% confidence intervals, means with standard errors, and quantiles.

### Statistical Modeling

We used GLMMs to quantify changes in (a) juvenile mosquito catch (number of larvae caught in SBSs) and (b) adult *Aedes* emergence (number of adults emerging from SBSs) following PPF dissemination. The Akaike information criterion (AIC) and the Bayesian information criterion (BIC) [30,31] unambiguously selected the negative binomial error structure (versus Poisson) as the best fit for our count data (see S1 Table). Our GLMMs accounted for unequal sampling effort due to missing SBS surveillance data by including the (log)number of operational SBSs in each dwelling each month as an offset. Since repeated observations were made over time in each dwelling, we specified dwelling ID as a random factor. Six dwelling-months produced no data (closed dwellings) and were excluded from the analyses, for a total of 2,294 SBS surveillance data points clustered in 100 dwellings. We specified intervention as a factor, indexing four consecutive periods: (1) before the intervention, or baseline; (2) citywide PPF dissemination (with some operational failures as noted above); (3) focal PPF dissemination (also with some operational failures); and (4) after PPF dissemination. We also tested alternative models excluding intervention effects (“null” models) or specifying, for each dwelling and month, (a) whether PPF dissemination (including DS maintenance) had/had not taken place at least once in the previous month (coded 1/0) or (b) the intensity/quality of dissemination, with 0 = no dissemination, 1 = unsupervised dissemination with possible operational failures, and 2 = supervised dissemination. For the second variable, for each month we summed the scores of the two fortnightly dissemination/maintenance events of the previous month, so this variable could take on integer values from 0 (no events) to 4 (two supervised events) (see S1 Data and S1 Fig). Dissemination/maintenance was recorded at the city sector level during citywide PPF dissemination and at the dwelling level during focal PPF dissemination. All these alternative GLMMs had, however, much larger AIC and BIC scores (consistently >50 units; see S1 Table) than the basic four-period models, on which we therefore base inference [30,31]. Our models controlled for the effects of rainfall (monthly total) and temperature (monthly average of maximum daily values); since these covariates were correlated (Pearson’s ρ = −0.704), we fit separate GLMMs adjusting for (standardized) rainfall and temperature. The Brazilian National Institute of Meteorology (INMET), which operates a meteorological station at the study locality, provided daily weather data (see S1 Data). GLMMs were fit using package lme4 1.1–10 in R 3.1.2 [32,33]. See S1 Table for details on the structure and relative performance of the full set of models used in each analysis, and S1 Text for a brief description of our original statistical modeling plan.

### Deterministic Modeling

Using our empirical data and a simple Ross-Macdonald–type model [20–22], we explored the potential effects of observed changes in *Aedes* spp. female emergence on pathogen transmission. We calculated the basic reproductive number, *R*_0_, for pathogens resembling *Aedes*-borne viruses, including dengue (see Table 1), and the ratio (denoted *m*) of female *Aedes* mosquitoes to susceptible humans, which was the parameter we aimed to affect with our intervention. *R*_0_ is given by

$$R_{0}=Da^{2}m\frac{e^{-\mu \tau }}{\mu }bc,$$

with parameters as defined in Table 1. We estimated monthly *m* ratios as the number of *Aedes* females emerging from SBSs each month in each dwelling divided by 4.5, the average number of people per dwelling in our study setting. We hence assumed that 100% of the local human population was susceptible to the pathogen, mirroring the current spread of Zika and chikungunya outside Africa [5–7]. To provide much more conservative estimates of intervention effects on *R*_0_, we repeated these analyses using three times as many emerging females as observed—i.e., using 3*m* instead of *m*. This represents the (unlikely) possibility that eight further breeding sites with mean productivity similar to that of our SBSs were present, on average, in each dwelling each month.

**Table 1:** Parameter values used to investigate the expected variation of the basic reproductive number, *R*_0_, of a mosquito-borne viral infection as a function of the ratio of emerging *Aedes* females to humans under five hypothetical scenarios.

Parameter	Symbol	Scenario				
		Optimistic	Fair/Realistic	Pessimistic	Gloomy	Worst Case
Infective period (days of viremia)	*D*	3	4	5	5	6
Extrinsic incubation period (days)	τ	8	7	6	7	6
Daily vector biting rate	*a*	0.7	0.8	0.9	1.0	1.0
Vector infectivity (probability per bite)	*b*	0.5	0.6	0.7	0.9	0.9
Human infectivity (probability per bite)	*c*	0.5	0.6	0.7	0.9	0.9
Daily vector death rate	μ	0.1	0.1	0.1	0.1	0.1
Vector:human ratio	*m*	Empirical monthly values				

## Results

### Descriptive Analyses

**Fig 2:**
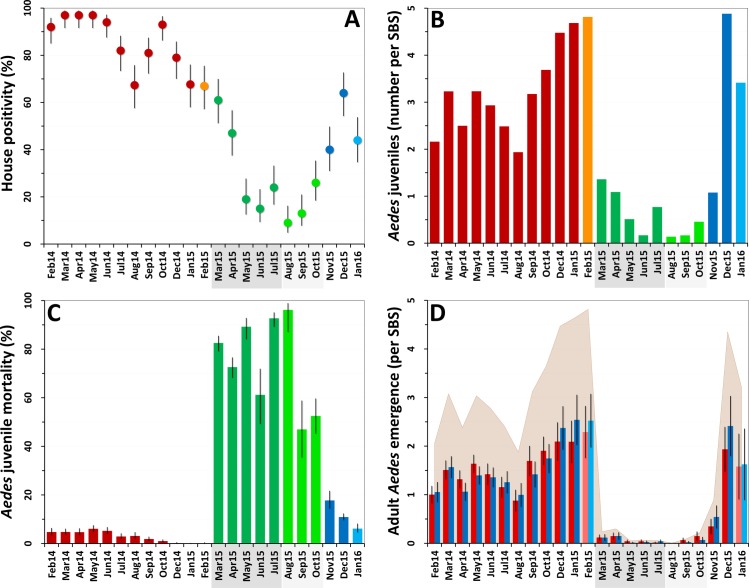
Changes in mosquito population metrics following deployment of mosquito-disseminated pyriproxyfen: descriptive graphs. (A) Monthly dwelling infestation by *Aedes* spp. (percent of dwellings in which at least one *Ae*. *albopictus* or *Ae*. *aegypti* juvenile was present in sentinel breeding sites [SBSs]); error bars are score 95% confidence intervals. (B) Mean monthly numbers of *Aedes* juveniles per SBS. (C) Monthly *Aedes* juvenile mortality (overall percent of juveniles that died before reaching adulthood); error bars are score 95% confidence intervals. (D) Mean monthly adult *Aedes* emergence (number of juvenile *Aedes* that developed into adults in each SBS); error bars are two standard errors. In all panels, the periods of citywide (dark grey) and focal (light grey) pyriproxyfen (PPF) dissemination are highlighted on the *x*-axes. Color coding in (A–C): red, pre-intervention (baseline) period, with orange indicating that just one survey was conducted in February 2015; dark green, citywide PPF dissemination; light green, focal PPF dissemination; blue, post-intervention period, with light blue indicating that just one survey was conducted in January 2016. Color coding in (D): red, females; blue, males; shaded area, total adult emergence; lighter red/blue, single-survey months (February 2015 and January 2016).

*Ae*. *albopictus* was the dominant mosquito species at the study site; overall, we caught 12,817 *Ae*. *albopictus* and 5,346 *Ae*. *aegypti* juveniles in our SBSs. House infestation by *Aedes* spp. fell from monthly values consistently about 70%–90% at baseline (mean 84.5%, median 87%, range 67%–97%) to a mean of 33% during citywide PPF dissemination (median 24%, range 15%–61%) and to a lowest value of 9% in the first month of focal dissemination (mean 16%, median 13%, range 9%–26%); afterwards, infestation gradually recovered to baseline values (Fig 2A). We also collected 58 *Culex* spp. (*Cx*. *quinquefasciatus* and a few *Cx*. *nigripalpus*) and 1,213 *Limatus* spp. (mainly *L*. *durhami*) larvae during the trial. House infestation by *Culex* spp. was consistently low before dissemination (median 1%, range 0%–6%); afterwards, just four dwellings were positive in just one month (April 2015). Dwelling infestation by *Limatus* spp. was recorded only before PPF dissemination (median 17.6%, range 0%–83%).

Juvenile *Aedes* catch fell from a median value of 3.20 individuals per SBS per month before the intervention (range 1.94–4.82, mean 3.28) to less than one juvenile per SBS per month during citywide (median 0.77, range 0.17–1.36, mean 0.78) and focal (median 0.17, range 0.14–0.46, mean 0.26) PPF dissemination. *Aedes* catch rose back to a mean of more than three larvae per SBS over the last 3 mo of the trial (Fig 2B). At the dwelling level, these figures translate into typical mean catches of about 7–17 juvenile *Aedes* per month before PPF dissemination, falling to a minimum of 0.52 (52 *Aedes* juveniles in 100 dwellings) in the first month of focal dissemination. Mean monthly catch per dwelling was 1.01 for *Limatus* spp. and 0.04 for *Culex* spp. before dissemination; except for seven *Culex* larvae caught in the second month of focal dissemination, neither genus appeared in samples taken during or after the intervention.

Before PPF dissemination, most *Aedes* juveniles survived to adulthood in our SBSs. Mean baseline monthly mortality was 1.9% (median 2.4%, range 0.0%–3.8%) for *Ae*. *albopictus* and 6.6% (median 5.5%, range 0.0%–17.8%) for *Ae*. *aegypti*. Monthly *Aedes* spp. mortality soared to 79.7% on average (range 61.2%–92.7%) during citywide PPF dissemination and reached a peak value of 96.2% (95% CI 87.0%–98.9%) in the first month of focal dissemination (Fig 2C). We could not investigate possible changes in juvenile *Limatus* mortality (mean at baseline 3.95%) because no larvae were caught after dissemination started. All *Culex* spp. juveniles caught before, but just three of seven caught during, PPF dissemination survived to adulthood.

The combined effects of much lower juvenile mosquito catches (Fig 2B) and much higher juvenile mortality (Fig 2C) yielded a striking citywide decrease of adult mosquito emergence during PPF dissemination (Fig 2D). Mean monthly *Aedes* adult emergence from SBSs was 1,077 (median 1,034, range 653–1,635) at baseline, for a mean of 3.2 adults per SBS per month (median 3.1, range 1.9–4.8) and 10.8 adults per dwelling per month (median 10.3, range 6.7–16.5). During citywide PPF dissemination, monthly emergence fell about 40-fold to just 56 adults on average (median 26, range 21–117), or 0.14 adults per SBS (median 0.07, range 0.06–0.30) and 0.56 adults per dwelling (median 0.26, range 0.21–1.17). Comparing extreme values (1,635 adults in January 2015 versus 21 adults in May 2015), adult *Aedes* emergence fell about 80-fold during citywide PPF dissemination. Further decreases were recorded during focal dissemination, down to a minimum of just two adult *Aedes* in total (a male and a female *Ae*. *albopictus*) emerging from SBSs, each in a different dwelling, in August 2015—an 800-fold reduction relative to January 2015. As with other metrics, adult *Aedes* emergence rose back to baseline values after PPF dissemination stopped (Fig 2D).

**Fig 3:**
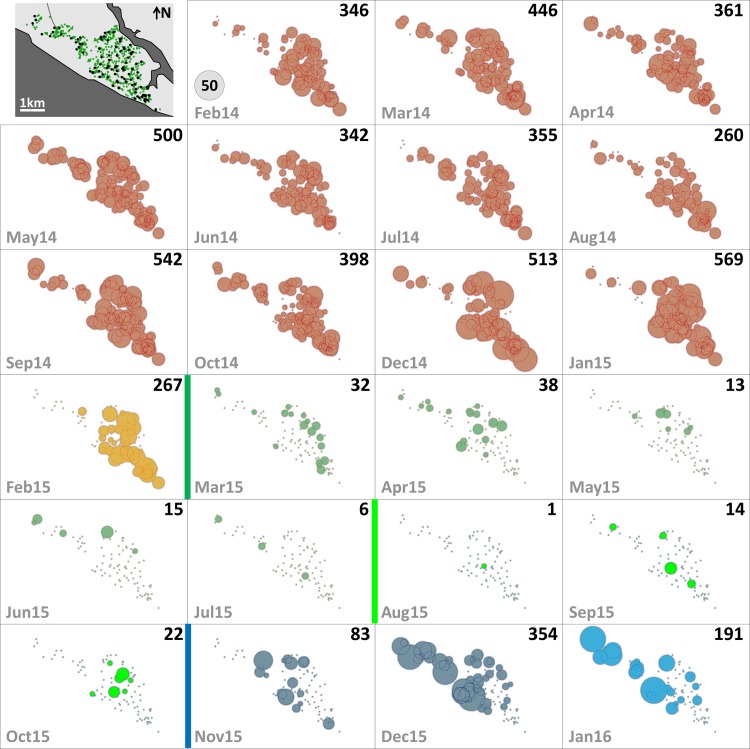
Monthly female *Aedes albopictus* emergence in each of the 100 surveillance dwellings. The distribution of dwellings (black dots) and pyriproxyfen dissemination stations (green dots) is shown in the first panel, where dots are overlaid on a schematic of Manacapuru. In the remaining panels, bubble size is proportional to the number of emerging *Ae*. *albopictus* females; the scale is shown as a grey bubble in the second panel. For each month, the total number of emerging *Ae*. *albopictus* females is shown in the upper right corner of the panel. Color coding: brown, pre-intervention (baseline) period, with yellow indicating a single-survey month; dark green, citywide PPF dissemination; light green, focal PPF dissemination; blue, post-intervention period, with light blue indicating a single-survey month. Temporal boundaries between periods are highlighted by colored vertical bars.

**Fig 4:**
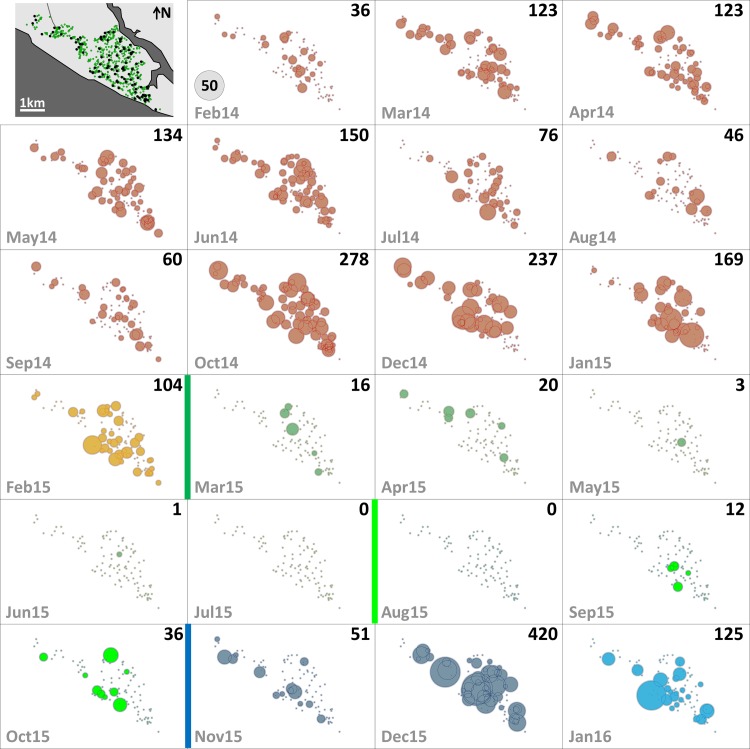
Monthly female *Aedes aegypti* emergence in each of the 100 surveillance dwellings. The distribution of dwellings (black dots) and pyriproxyfen dissemination stations (green dots) is shown in the first panel, where dots are overlaid on a schematic of Manacapuru. In the remaining panels, bubble size is proportional to the number of emerging *Ae*. *aegypti* females; the scale is shown as a grey bubble in the second panel. For each month, the total number of emerging *Ae*. *aegypti* females is shown in the upper right corner of the panel. Color coding: brown, pre-intervention (baseline) period, with yellow indicating a single-survey month; dark green, citywide PPF dissemination; light green, focal PPF dissemination; blue, post-intervention period, with light blue indicating a single-survey month. Temporal boundaries between periods are highlighted by colored vertical bars.

Since mosquito females but not males transmit human pathogens, we separately assessed *Aedes* female emergence from our SBSs. Table 2 summarizes monthly female emergence, and Figs 3 and 4 show, respectively, the numbers of *Ae*. *albopictus* and *Ae*. *aegypti* females emerging from SBSs in each dwelling and month. Monthly *Aedes* female emergence fell from an average of 536.6 (median 530, range 306–750) before to 28.8 (median 16, range 6–58) during citywide PPF dissemination; median values were therefore 33-fold lower, and extreme values 125-fold lower, during than before citywide dissemination. Again, this reduction became even larger over the focal dissemination period, with just one *Aedes* female emerging from the SBSs in August 2015—a >500-fold decrease compared to January 2015 (Table 2; Figs 2D, 3 and 4).

**Table 2:** Monthly female *Aedes* spp. emergence from sentinel breeding sites set in 100 surveillance dwellings, Manacapuru, Amazonas, Brazil, February 2014–January 2016.

Pyriproxyfen Dissemination Period	Month	Number of Dwellings	Number of SBSs	Number of *Aedes* Mosquitoes Emerging				
				*Ae*. *albopictus*	*Ae*. *aegypti*	Total *Aedes*	Per SBS	Per Dwelling
**Before**	Feb 2014	100	381	346	36	382	1.00	3.82
** **	Mar 2014	100	377	446	123	569	1.51	5.69
** **	Apr 2014	100	367	361	123	484	1.32	4.84
** **	May 2014	100	387	500	134	634	1.64	6.34
** **	Jun 2014	100	346	342	150	492	1.42	4.92
** **	Jul 2014	100	373	355	76	431	1.16	4.31
** **	Aug 2014	98	348	260	46	306	0.88	3.12
** **	Sep 2014	100	355	542	60	602	1.70	6.02
** **	Oct 2014	100	355	398	278	676	1.90	6.76
** **	Dec 2014	100	358	513	237	750	2.09	7.50
** **	Jan 2015	99	353	569	169	738	2.09	7.45
** **	Feb 2015*	97	162	267	104	371	2.29	3.82
**Citywide**	Mar 2015	100	400	32	16	48	0.12	0.48
** **	Apr 2015	100	392	38	20	58	0.15	0.58
** **	May 2015	100	381	13	3	16	0.04	0.16
** **	Jun 2015	100	398	15	1	16	0.04	0.16
	Jul 2015	100	391	6	0	6	0.02	0.06
**Focal**	Aug 2015	100	376	1	0	1	0.003	0.01
	Sep 2015	100	395	14	12	26	0.07	0.26
	Oct 2015	100	392	22	36	58	0.15	0.58
**After**	Nov 2015	100	387	83	51	134	0.35	1.34
	Dec 2015	100	400	354	420	774	1.94	7.74
	Jan 2016*	100	200	191	125	316	1.58	3.16

The target number of operational sentinel breeding sites (SBSs) was four per dwelling per month. The target number of surveillance dwellings with data was 100 per month.

*Only one survey was conducted in February 2015, when pyriproxyfen dissemination stations were deployed citywide, and in January 2016, when the trial ended.

### Statistical Modeling

**Fig 5:**
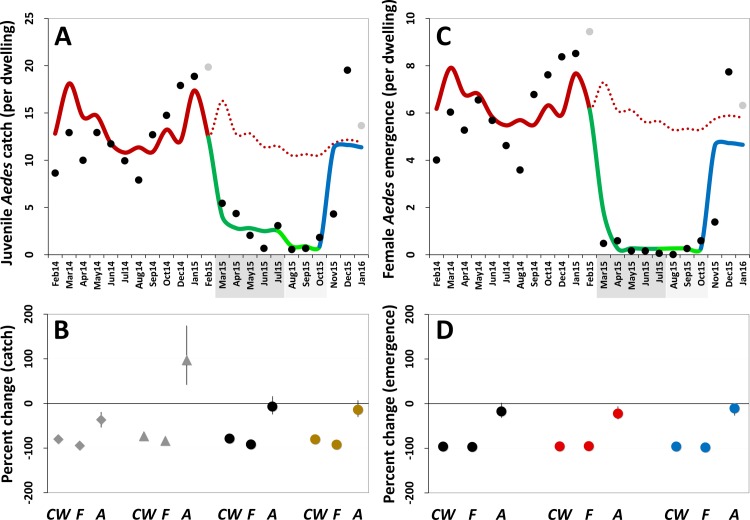
Estimated impact of mosquito-disseminated pyriproxyfen on *Aedes* populations: results of generalized linear mixed models. (A) Mean monthly *Aedes* juvenile catch per dwelling: dots, observed values (in grey, values adjusted for single-survey months); solid line, model predictions (red, pre-intervention period; dark green, citywide pyriproxyfen [PPF] dissemination; light green, focal PPF dissemination; blue, post-intervention period); dotted line, model-predicted trajectory in the absence of intervention as a function of monthly rainfall and adjusted for the number of operational sentinel breeding sites and dwelling-level clustering. (B) Model-predicted percent change (with 95% confidence intervals) in juvenile mosquito catch relative to the pre-intervention period (*CW*, citywide PPF dissemination; *F*, focal dissemination; *A*, after PPF dissemination): diamonds, *Ae*. *albopictus*; triangles, *Ae*. *aegypti*; black circles, *Aedes* spp.; gold circles, all mosquito species. (C) Mean monthly *Aedes* adult emergence per dwelling, with coding as in (A). (D) Model-predicted percent change (with 95% confidence intervals) in adult *Aedes* emergence relative to the pre-intervention period, with periods coded as in (B); black circles, all *Aedes* adults; red circles, *Aedes* females; blue circles, *Aedes* males. In (A) and (C), the periods of citywide (dark grey) and focal (light grey) PPF dissemination are highlighted on the *x*-axes.

GLMMs estimated strong negative effects of PPF dissemination on juvenile mosquito catch (Table 3; Fig 5A). Compared with baseline values, mean juvenile catch (all species) was estimated to fall by 80.2% (95% CI 76.3%–83.5%) and 92.1% (95% CI 89.9%–94.0%) during, respectively, citywide and focal PPF dissemination. The largest effect estimate was for *Ae*. *albopictus* catch, with a 94.1% reduction (95% CI 92.0%–95.6%) during focal dissemination and with negative effects still evident after dissemination stopped (36.2% reduction, 95% CI 18.9%–53.7%) (Fig 5B). *Ae*. *aegypti* mean catch was estimated to fall by 72.7% (95% CI 63.9%–79.4%) and 83.1% (95% CI 74.8%–88.7%) during citywide and focal dissemination, respectively (Fig 5B); the estimated increase in *Ae*. *aegypti* catch after PPF dissemination (Fig 5B) is driven by four outlier dwellings with a mean monthly catch of 30.2 *Ae*. *aegypti* juveniles per SBS (see S2 Fig).

**Table 3:** Estimated effects of mosquito-disseminated pyriproxyfen on juvenile mosquito catch: results from generalized linear mixed models.

Mosquito	Term	Estimate	Standard Error	95% CI	
				Upper Bound	Lower Bound
***Ae*. *albopictus***	**Intercept**	0.82	0.06	0.71	0.94
	**Period**				
	Before PPF dissemination	Ref.			
	Citywide PPF dissemination	−1.60	0.10	−1.80	−1.41
	Focal PPF dissemination	−2.83	0.15	−3.12	−2.53
	After PPF dissemination	−0.45	0.12	−0.77	−0.21
	**Rainfall**	0.16	0.04	0.08	0.24
	**Dwelling random effect (SD)**	0.32		0.20	0.44
***Ae*. *aegypti***	**Intercept**	−0.32	0.09	−0.49	−0.15
	**Period**				
	Before PPF dissemination	Ref.			
	Citywide PPF dissemination	−1.30	0.14	−1.58	−1.02
	Focal PPF dissemination	−1.78	0.20	−2.18	−1.38
	After PPF dissemination	0.68	0.17	0.35	1.01
	**Rainfall**	0.15	0.07	0.02	0.28
	**Dwelling random effect (SD)**	0.36		0.17	0.54
***Ae*. *albopictus* + *Ae*. *aegypti***	**Intercept**	1.13	0.06	1.03	1.24
	**Period**				
	Before PPF dissemination	Ref.			
	Citywide PPF dissemination	−1.54	0.09	−1.71	−1.36
	Focal PPF dissemination	−2.48	0.13	−2.74	−2.22
	After PPF dissemination	−0.07	0.11	−0.28	0.15
	**Rainfall**	0.15	0.04	0.08	0.23
	**Dwelling random effect (SD)**	0.28		0.18	0.39
***Aedes* + *Culex* + *Limatus***	**Intercept**	1.21	0.05	1.10	1.32
	**Period**				
	Before PPF dissemination	Ref.			
	Citywide PPF dissemination	−1.62	0.09	−1.80	−1.44
	Focal PPF dissemination	−2.54	0.13	−2.81	−2.29
	After PPF dissemination	−0.15	0.11	−0.36	0.07
	**Rainfall**	0.18	0.04	0.10	0.25
	**Dwelling random effect (SD)**	0.29		0.19	0.40

See Akaike and Bayesian information criterion values in S1 and S2 Tables.

PPF, pyriproxyfen; SD, standard deviation.

Mean reductions in adult *Aedes* emergence relative to baseline, as estimated by a GLMM (Table 4), were 96.0% (95% CI 95.2%–96.8%) during citywide and 96.8% (95% CI 95.8%–97.6%) during focal PPF dissemination, with emergence rising back to baseline values after dissemination ended (17.3% mean reduction relative to baseline but with a 95% CI ranging from a 31.6% decrease to a 2.0% increase). For *Aedes* females, GLMM estimates suggest emergence reductions of 95.6% (95% CI 94.6%–96.5%) during citywide and 95.1% (95% CI 93.4%–96.3%) during focal dissemination, with emergence still 22.1% (95% CI 5.8%–34.9%) lower in the post-intervention period than at baseline (Table 4; Fig 5C and 5D). Results were similar for *Aedes* males, with a maximum estimated reduction in emergence of 98.0% (95% CI 97.1%–98.7%) in the focal dissemination period (Table 4; Fig 5D).

**Table 4:** Estimated effects of mosquito-disseminated pyriproxyfen on adult *Aedes* emergence: results from generalized linear mixed models.

Sex	Term	Estimate	Standard Error	95% CI	
				Upper Bound	Lower Bound
**Females *+* males**	**Intercept**	1.10	0.06	0.99	1.21
	**Period**				
	Before PPF dissemination	Ref.			
	Citywide PPF dissemination	−3.23	0.10	−3.43	−3.03
	Focal PPF dissemination	−3.45	0.15	−3.74	−3.16
	After PPF dissemination	−0.19	0.10	−0.38	0.02
	**Rainfall**	0.12	0.04	0.05	0.20
	**Dwelling random effect (SD)**	0.37		0.27	0.47
**Females**	**Intercept**	0.40	0.05	0.30	0.50
	**Period**				
	Before PPF dissemination	Ref.			
	Citywide PPF dissemination	−3.13	0.11	−3.35	−2.92
	Focal PPF dissemination	−3.01	0.15	−3.31	−2.72
	After PPF dissemination	−0.25	0.10	−0.43	−0.06
	**Rainfall**	0.11	0.04	0.04	0.18
	**Dwelling random effect (SD)**	0.30		0.20	0.40
**Males**	**Intercept**	0.39	0.06	0.27	0.50
	**Period**				
	Before PPF dissemination	Ref.			
	Citywide PPF dissemination	−3.20	0.12	−3.43	−2.97
	Focal PPF dissemination	−3.92	0.20	−4.33	−3.54
	After PPF dissemination	−0.11	0.10	−0.31	−0.08
	**Rainfall**	0.11	0.04	0.04	0.18
	**Dwelling random effect (SD)**	0.39		0.30	0.50

See Akaike and Bayesian information criterion values in S1 and S3 Tables.

PPF, pyriproxyfen; SD, standard deviation.

All the above intervention effect estimates were fully consistent with those derived from models in which we used temperature instead of rainfall to provide adjustment for weather conditions; as expected, our GLMMs overall suggest moderate positive effects of rainfall and weaker negative effects of maximum temperature on mosquito population metrics (see S2 and S3 Tables).

### Deterministic Modeling

**Fig 6:**
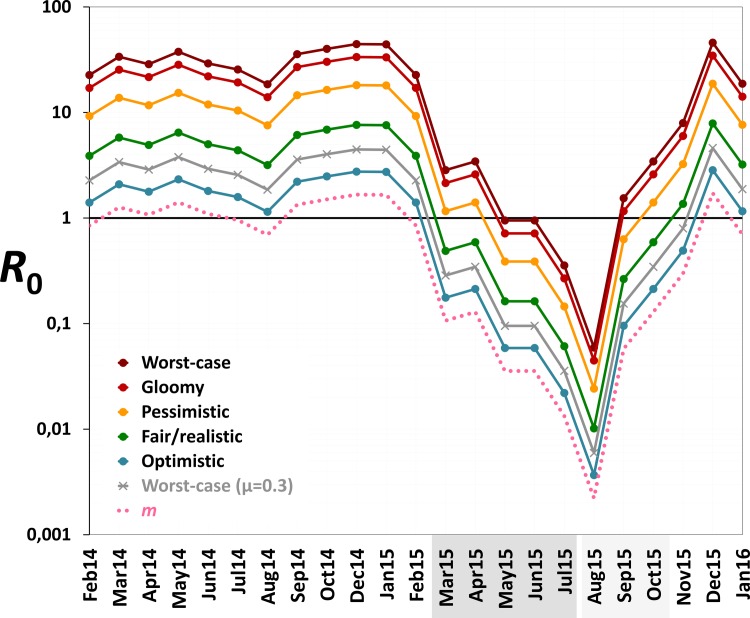
Monthly estimates of the basic reproductive number (*R*_0_) of mosquito-borne viruses similar to dengue, Zika, or chikungunya. We considered scenarios ranging from optimistic to very adverse (see parameter values for each scenario in Table 1); the grey line corresponds to the worst-case scenario but with a higher value of the mean daily female mosquito death rate (*μ* = 0.3 instead of 0.1) to approximate data from wild *Ae*. *aegypti* populations (see [34–37]). The pink dotted line shows empirical monthly values of the number of *Aedes* females per person (parameter *m*) in our study setting and period. The periods of citywide (dark grey) and focal (light grey) PPF dissemination are highlighted on the *x*-axis.

Monthly values of *m*, an estimate of the mean number of *Aedes* females emerging per person, fell from 1.2 (median 1.2; range 0.7–1.7) before PPF dissemination to 0.06 during both citywide (median 0.04, range 0.01–0.13) and focal (median 0.06, range 0.002–0.13) PPF dissemination. Fig 6 shows *R*_0_ values as a function of monthly *m* ratios in five scenarios ranging from optimistic to worst case (see Table 1). Recall that *R*_0_ measures the number of new infections arising from a primary case [20–22], so an infection can persist in a host population only if *R*_0_ > 1.0; recall also that our models assume a naïve human population with no immunity against the pathogen. Fig 6 shows that, across scenarios, the reduction of monthly *m* ratio seen during PPF dissemination is predicted to consistently bring *R*_0_ to <1.0, whereas baseline *m* values (first 12 mo) predict *R*_0_ values typically between 2.0 (optimistic scenario; range 1.15–2.75) and 5.5 (fair/realistic scenario; 3.18–7.63). Even in the worst-case scenario, with baseline *R*_0_ = 32 (range 19–45), the intervention would bring *R*_0_ to <1.0 for 4 and 9 mo assuming, respectively, daily vector death rates of *μ* = 0.1 and *μ* = 0.3 (see [34–37]) (Fig 6). A reanalysis using three times as many emerging *Aedes* females as observed predicts *R*_0_ < 1.0 for 1 mo (worst-case scenario, with baseline *R*_0_ from 56 to 133) to 8 mo (optimistic scenario, with baseline *R*_0_ ranging from 3 to 8) (see S3 Fig).

## Discussion

In this study we have shown that a sharp citywide decrease in mosquito vector populations followed the application of a low-technology tactic based on mosquito-disseminated PPF in a tropical town. Population declines were observed for *Aedes* and *Culex* spp., two foremost vectors of human disease, and for *Limatus* spp. The 95%–96% reduction in *Aedes* female emergence we report has the potential of blocking arbovirus transmission under scenarios ranging from somewhat optimistic to overtly adverse. The control of urban *Culex* spp. could have similar effects on the spread of important pathogens ranging from West Nile virus to lymphatic filariae; *Culex* spp. might in addition transmit Zika virus [38], although this is yet to be confirmed. Suppression of urban *Limatus* populations has less clear public health implications, but several bunyaviruses capable of infecting mammals have been isolated from mosquitoes of this day-biting genus in Amazonia [39].

Our findings suggest that mosquito-disseminated PPF could be particularly relevant for the control of epidemic outbreaks such as those seen when Zika, dengue, or chikungunya virus sweeps through immunologically naïve populations. Given partial herd immunity, the more stable endemic-epidemic transmission of, for example, dengue in many countries [40] would be even easier to interrupt. We note, in addition, that some of the parameter values used in our calculations (Table 1) probably exceed typical real values; in fact, our baseline *R*_0_ estimates are higher than those reported for dengue epidemics in Brazil [34]. Daily death rates of *Aedes* females, for example, have been estimated as *μ* ≈ 0.2–0.4 in Brazil and Puerto Rico [35–37]. If, moreover, PPF reduces the lifespan of female *Aedes* as it does with *Anopheles gambiae* [41], this would further increase *μ*. Using *μ* = 0.3 instead of 0.1, our models suggest that *R*_0_ would be brought to <1.0 for 9 mo under the worst-case scenario—and for 6 mo even assuming three times as many emerging females as observed (Figs 6 and S3). Further, *Aedes* infectivity (parameter *b* in Table 1) is probably lower, on average, than we assumed in our calculations (see, e.g., [42,43] for Zika virus). Thus, in general, our models likely underestimate the potential intervention effects on *R*_0_. This suggests that mosquito-disseminated PPF might block arbovirus transmission citywide even under very adverse circumstances—an entirely susceptible population, long-lasting viremias, frequent mosquito biting, short extrinsic incubation periods, and high probabilities of virus transmission from vector to human and vice versa (Table 1; Figs 6 and S3).

The findings we report come, however, with several important caveats. The most obvious is that ours was a before–after, single-site trial lacking independent replicates, which limits the strength of the evidence we present. To partly mitigate this limitation, we estimated intervention effects using detailed 12-mo baseline data, and accounted for dwelling-level repeated measures, weather conditions, and unequal sampling effort using GLMMs. The size, sign, and consistency of effect estimates—which fully align with expectations based on mosquito biology and previous reports of PPF effects [26,27,29,44]—reinforce our confidence in the outcome of our analyses. In addition, that our findings are consistent with results from a neighborhood in a different city [29] suggests that PPF dissemination effects might be replicable elsewhere. Control results might however depend on the local availability of alternative breeding sites, which we did not measure. The impact of the intervention could thus be reduced in places where competing larval habitats are widespread enough to distract egg-laying females away from DSs. A second key limitation is that our SBS data are difficult to interpret in terms of true adult mosquito density or abundance [45]. While this should not greatly affect our estimates of *relative* change in juvenile catch and adult emergence, it calls for a cautious reading of our *R*_0_ results. Even if our complementary analyses using three times as many emerging females as observed are reassuring, we regard our *R*_0_ estimates as explicit, plausible hypotheses to be tested in future trials—ideally, cluster-randomized trials with replicate intervention and control sites and including a blind prospective assessment of arboviral infection incidence [46]. Finally, we note that we did not have the means to measure PPF in our SBSs, and therefore lack direct evidence of PPF dissemination. However, we did not record any extreme weather event or vector control intervention that could account for our observations. Further, alternative GLMMs investigating dissemination intensity/quality (coded as a 1-mo-lagged 0–4 variable; see Methods) suggested “dose-dependent” effects—with, for instance, a 57.1% (95% CI 54.3%–59.8%) reduction in monthly adult *Aedes* emergence for each unit increase in dissemination intensity/quality (see S4 Fig). In sum, we are confident that the striking changes in mosquito demographics we report were real and were a direct consequence of our intervention. The caveats discussed above call, however, for a cautious interpretation of our results, particularly regarding virus transmission—which we did not measure empirically.

In our trial, local vector control staff deployed and maintained PPF DSs, with the research team providing initial training and nearly continuous supervision—in which we monitored, but did not interfere with, PPF dissemination or DS maintenance. This led to some operational problems, including failure to maintain or deploy some DSs as scheduled (mainly due to lack of fuel for reaching the more distant northwestern city sector) and suboptimal PPF grinding (see S1 Data and S1 Fig). Model comparisons showed, however, that four-intervention-period GLMMs performed much better (as measured by much lower AIC and BIC scores [30,31]) than alternative GLMMs with more detailed descriptions of PPF dissemination dynamics (S1 Table). This suggests that operational problems had little impact on overall intervention effects, and hence that the strategy may work under the constraints of real-life vector control efforts. In practical terms, the most relevant obstacle was that the PPF we used is formulated as coarse sand-like granules that had to be manually ground to talc-like powder; this was time-consuming and yielded dust particles of variable, unknown size. In preparation for larger-scale trials, we are using mechanical micronizers to get PPF dust of standardized particle size.

One additional asset of our approach is that it may easily be combined with other interventions, traditional or novel, in integrated mosquito control strategies. For example, control agents and community members could focus on treating or destroying large, conspicuous, and accessible breeding sites, while mosquitoes disseminate PPF to the small, cryptic, and inaccessible larval habitats often used by *Aedes* spp. The community could engage in DS maintenance with support from local health agents; this would empower communities and may enhance acceptability while reducing costs. During outbreaks, indoor insecticide spraying could synergize PPF dissemination to quickly block transmission; in sites with effective early warning systems, spatially targeted interventions could be deployed as soon as the first cases of infection (in humans, vectors, or sentinel hosts) are detected. In general, flexible PPF dissemination strategies can be designed to suit particular needs in time and space. For example, focally deploying DSs at high densities could protect people in transmission-prone places such as hospitals, schools, stadiums, markets, churches, cemeteries, hotels, or prisons. Specific dissemination schemes in airports, bus/train stations, or ports (even on ships) might help limit man-mediated *Aedes* spread. Mosquito-disseminated PPF also holds promise for sites without mosquito-borne disease transmission but where mosquito bites cause skin lesions, allergies, distress, or economic losses (e.g., by affecting tourism). We also note that the 95%–98% reduction in adult *Aedes* emergence we recorded (Fig 5) could allow PPF dissemination to contribute to strategies based on the release of sterile, transgenic, or *Wolbachia*-transinfected mosquitoes, which require a high enough ratio of modified to wild mosquitoes [47–50]. The scale of mosquito releases (and associated costs including those of mass rearing) could be considerably reduced after a pulse of mosquito-disseminated PPF crashes local wild populations. Finally, we stress that combining PPF with products or tactics with different modes of action [46] would help reduce the odds of selecting resistant mosquitoes—a concern we also plan to address by testing larvicides other than PPF in experimental dissemination trials.

Here we have shown, in summary, that mosquito-disseminated PPF has the potential to become a major tool for urban mosquito control and, consequently, for the prevention of mosquito-borne diseases. These findings might be equally relevant for rapidly spreading emerging arboviral infections, including Zika and chikungunya, and for better-established endemic pathogens, including dengue, West Nile, and Japanese encephalitis viruses. Cluster-randomized, multi-site controlled trials are now necessary to provide stronger evidence for (or against) these hypotheses [46]. We plan to conduct one such trial in the context of the Brazilian dengue control program, which recently recommended considering our approach for inclusion in national guidelines [51]. Based on the present findings, we anticipate that randomized controlled trials will show that mosquito-disseminated PPF can develop into a new, crucial means for improving global public health.

## Supporting Information

S1 Data Raw data. Mosquito catch and emergence by species, plus intervention and weather covariate values.
(XLSX)

S1 Fig Pyriproxyfen dissemination in the vicinity of each surveillance dwelling. Each circle is centered on a surveillance dwelling, with circle size proportional to dissemination intensity/quality: two rounds of supervised dissemination (largest circles, value 4); one round of supervised dissemination and one round of unsupervised dissemination (value 3); one round of supervised dissemination (value 2); one round of unsupervised dissemination (value 1); or no dissemination (smallest circles, value 0). Dissemination was scheduled to be citywide in March–July 2015 and focal in August–October 2015. Note that dissemination failures mainly affected the northwestern sector of the town (the most distant from vector control headquarters), where three consecutive dissemination cycles (in May–June 2015) were not completed.
(PDF)

S2 Fig Monthly number of *Ae*. *aegypti* juveniles caught in each dwelling. Boxplots show the 10th, 25th, 50th, 75th, and 90th quantiles; note the four outliers in the last 2 mo of monitoring.
(PDF)

S3 Fig Monthly estimates of the basic reproductive number (*R*_0_) of mosquito-borne viruses similar to dengue, Zika, or chikungunya. We considered scenarios ranging from optimistic to very adverse (see parameter values for each scenario in Table 1) and used three times as many emerging females as observed in our study (i.e., 3*m* instead of *m*; pink dotted line); the grey line corresponds to the worst-case scenario but with a higher value of the mean daily female mosquito death rate (*μ* = 0.3 instead of 0.1) to approximate data from wild *Ae*. *aegypti* populations (see [34–37]).
(PDF)

S4 Fig Reduction in adult *Aedes* emergence (with 95% confidence intervals) as a function of pyriproxyfen dissemination intensity/quality (measured as a 0–4 score). Predictions from a generalized linear mixed model adjusting for monthly rainfall, the number of operational sentinel breeding sites, and dwelling-level clustering.
(PDF)

S1 Table The full set of generalized linear mixed models used in each analysis. Model structure and relative model performance, as measured through Akaike and Bayesian information criteria, are provided.
(XLSX)

S2 Table Juvenile mosquito catch: results of generalized linear mixed models with either rainfall or temperature as the weather covariate. Parameter estimates, standard errors, and values of the Akaike and Bayesian information criteria are provided.
(PDF)

S3 Table Adult *Aedes* emergence: results of generalized linear mixed models with either rainfall or temperature as the weather covariate. Parameter estimates, standard errors, and values of the Akaike and Bayesian information criteria are provided.
(PDF)

S1 Text Original plan for statistical modeling. (PDF)

## Abbreviations

AIC: Akaike information criterion
BIC: Bayesian information criterion
DS: dissemination station
GLMM: generalized linear mixed model
PPF: pyriproxyfen
SBS: sentinel breeding site
