# Supplementary figures and images for: Mosquito-Disseminated Insecticide for Citywide Vector Control and Its Potential to Block Arbovirus Epidemics: Entomological Observations and Modeling Results from Amazonian Brazil
Source: PLoS Med. 2017 Jan 17;14(1):e1002213. doi: 10.1371/journal.pmed.1002213 (PMC5240929; doi:10.1371/journal.pmed.1002213)

**March 15**

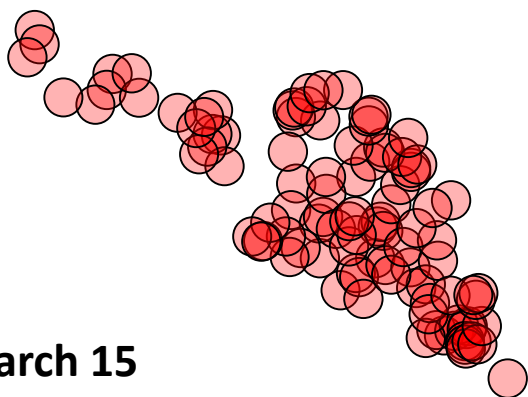

**April 15**

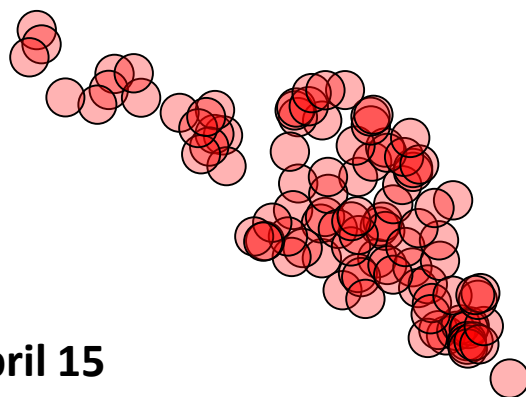

**May 15**

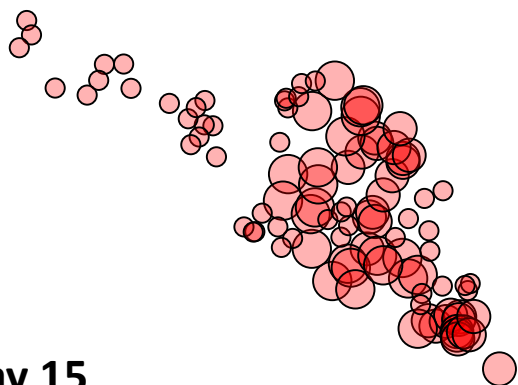

**June 15**

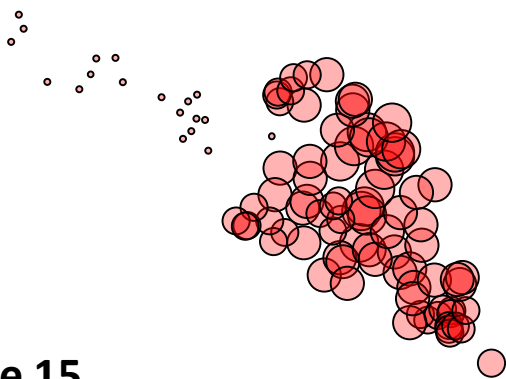

**July 15**

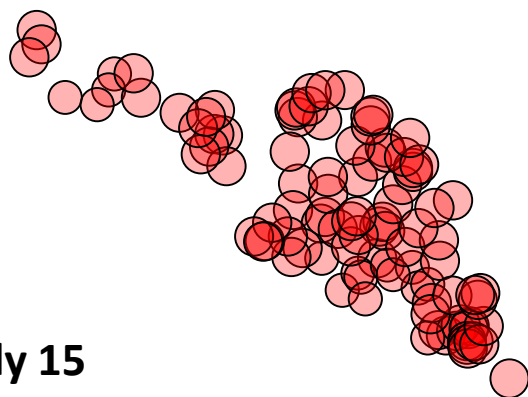

**August 15**

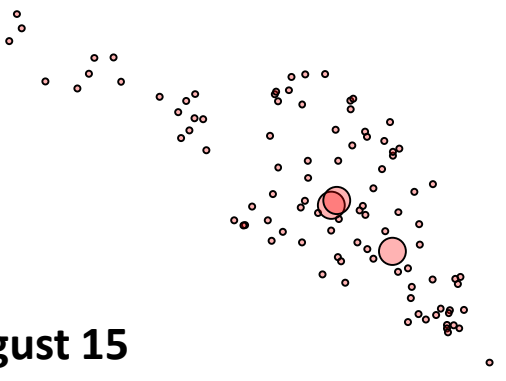

**September 15**

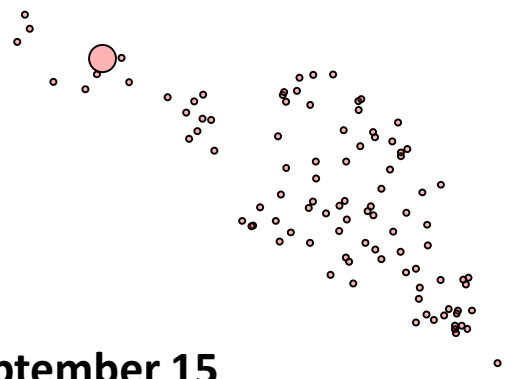

**October 15**

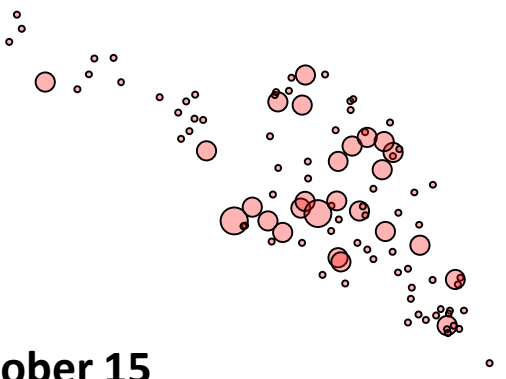

Supplement: S1 Fig — Each circle is centered on a surveillance dwelling, with circle size proportional to dissemination intensity/quality: two rounds of supervised dissemination (largest circles, value 4); one round of supervised dissemination and one round of unsupervised dissemination (value 3); one round of supervised dissemination (value 2); one round of unsupervised dissemination (value 1); or no dissemination (smallest circles, value 0). Dissemination was scheduled to be citywide in March–July 2015 and focal in August–October 2015. Note that dissemination failures mainly affected the northwestern sector of the town (the most distant from vector control headquarters), where three consecutive dissemination cycles (in May–June 2015) were not completed. (PDF) [file pmed.1002213.s002.pdf]

# *Aedes aegypti* juvenile catch

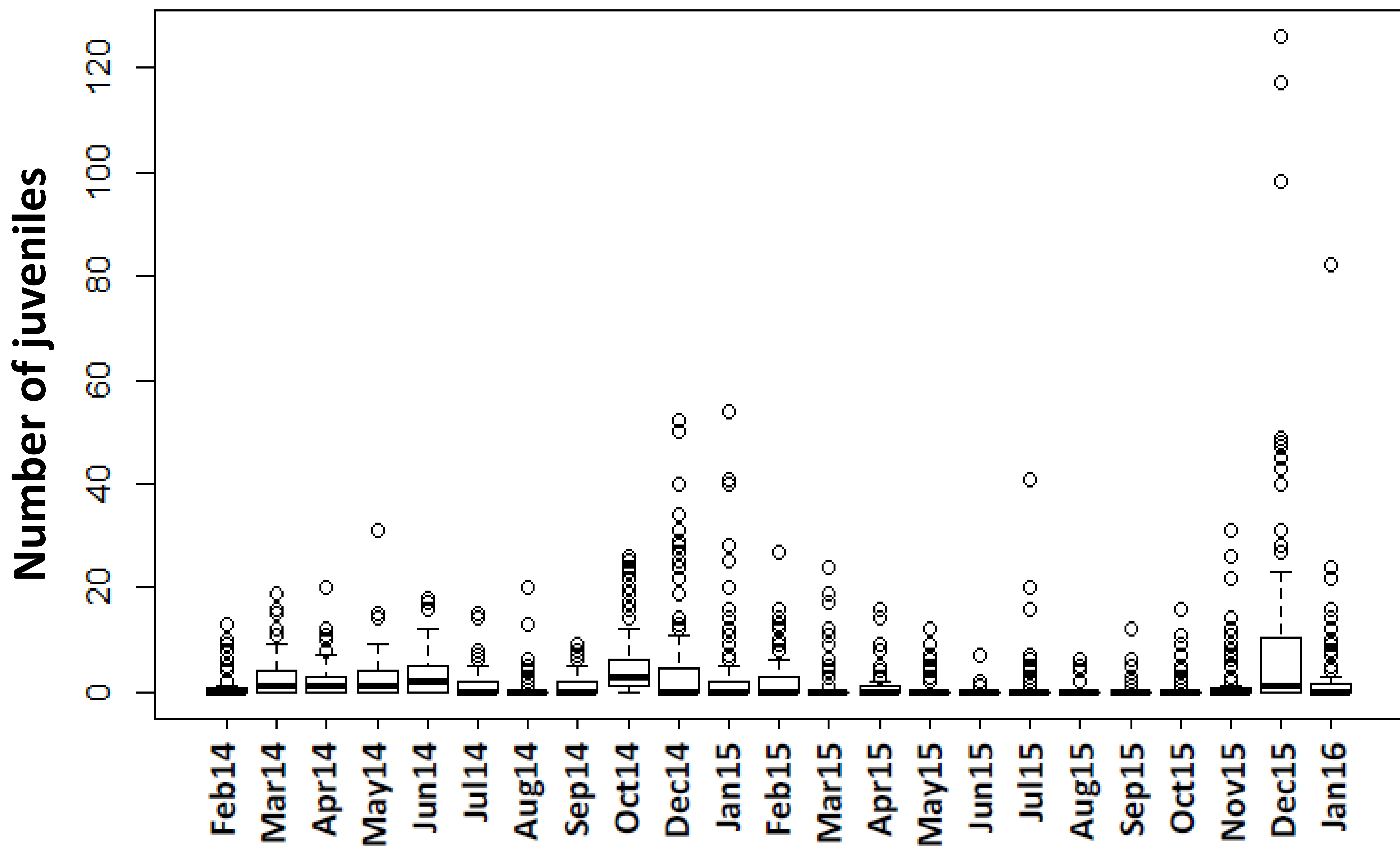

Supplement: S2 Fig — Boxplots show the 10th, 25th, 50th, 75th, and 90th quantiles; note the four outliers in the last 2 mo of monitoring. (PDF) [file pmed.1002213.s003.pdf]

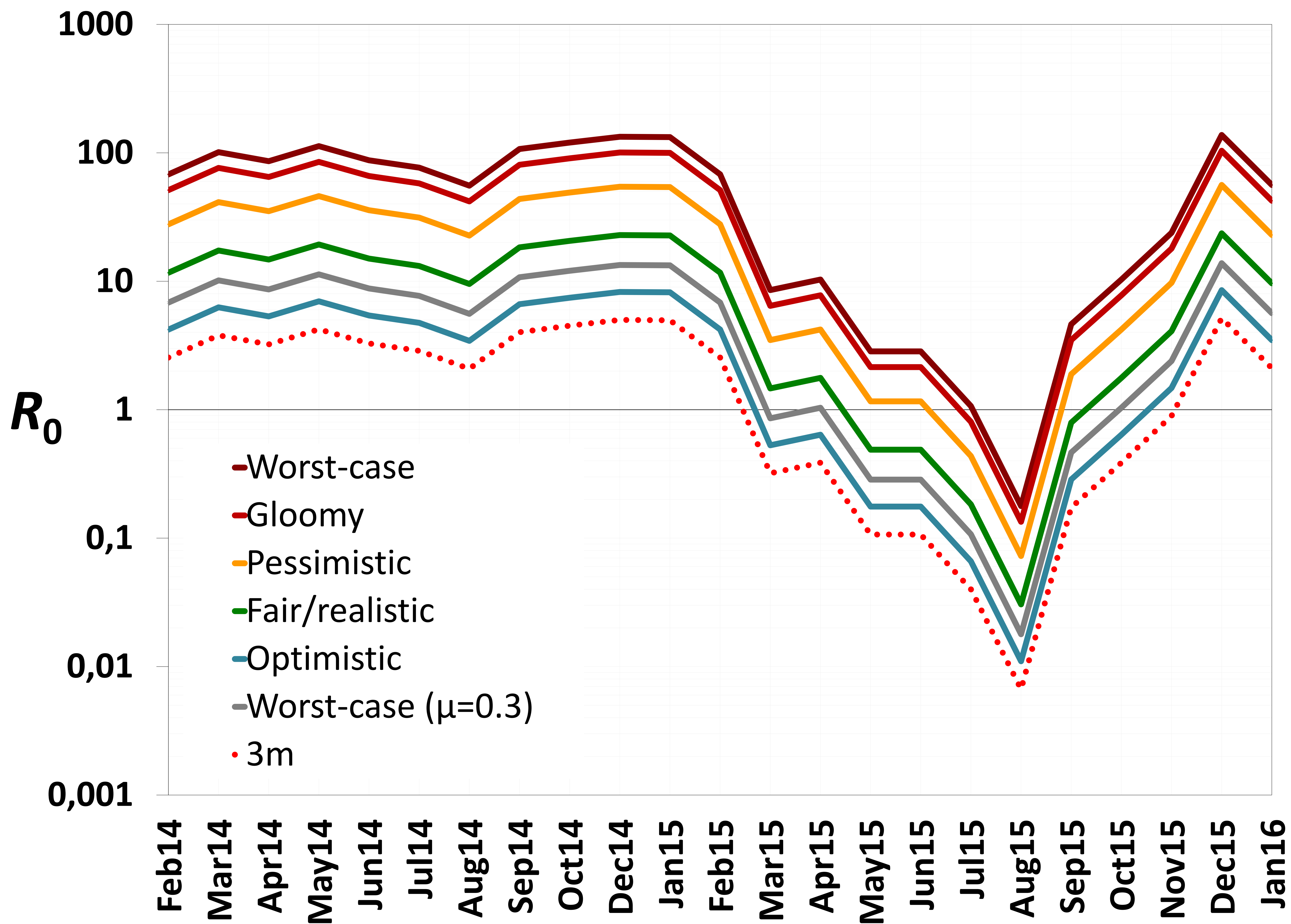

Supplement: S3 Fig — We considered scenarios ranging from optimistic to very adverse (see parameter values for each scenario in Table 1) and used three times as many emerging females as observed in our study (i.e., 3m instead of m; pink dotted line); the grey line corresponds to the worst-case scenario but with a higher value of the mean daily female mosquito death rate (μ = 0.3 instead of 0.1) to approximate data from wild Ae. aegypti populations (see [34–37]). (PDF) [file pmed.1002213.s004.pdf]

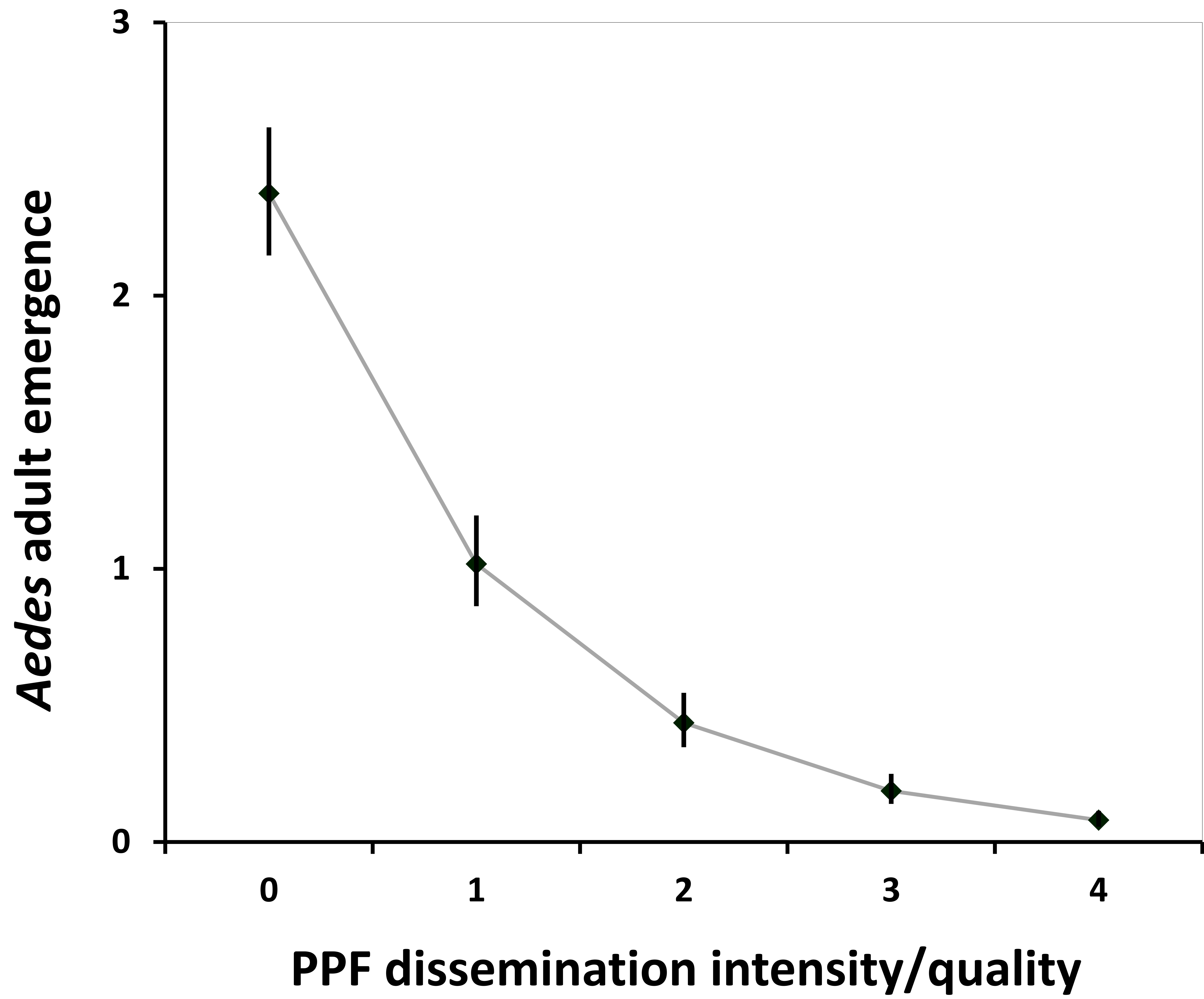

Supplement: S4 Fig — Predictions from a generalized linear mixed model adjusting for monthly rainfall, the number of operational sentinel breeding sites, and dwelling-level clustering. (PDF) [file pmed.1002213.s005.pdf]
