# Supplementary material for: Mosquito-Disseminated Insecticide for Citywide Vector Control and Its Potential to Block Arbovirus Epidemics: Entomological Observations and Modeling Results from Amazonian Brazil
Source: PLoS Med. 2017 Jan 17;14(1):e1002213. doi: 10.1371/journal.pmed.1002213 (PMC5240929; doi:10.1371/journal.pmed.1002213)
Supplement: S2 Table — Parameter estimates, standard errors, and values of the Akaike and Bayesian information criteria are provided. (PDF) [file pmed.1002213.s007.pdf]

S2 Table

S2 Table. Juvenile mosquito catch: results of generalized linear mixed models with either rainfall or temperature as the weather covariate

Mosquito	Rainfall models				Temperature models			
	Estimate	SE	AIC	BIC	Estimate	SE	AIC	BIC
Aedes albopictus			10,978	11,018			10,991	11,032
Intercept	0.82	0.06			0.83	0.06		
Period								
Before	Ref.				Ref.			
Citywide	−1.60	0.10			−1.57	0.10		
Focal	−2.83	0.15			−2.88	0.20		
After	−0.45	0.12			−0.47	0.13		
Rainfall	0.16	0.04						
Temperature					−0.07	0.06		
Dwelling (SD)	0.32				0.32			
Aedes aegypti			7101	7141			7102	7142
Intercept	−0.32	0.09			−0.37	0.10		
Period								
Before	Ref.				Ref.			
Citywide	−1.30	0.14			−1.31	0.15		
Focal	−1.78	0.20			−1.47	0.31		
After	0.68	0.17			0.79	0.19		
Rainfall	0.15	0.07						
Temperature					−0.23	0.11		
Dwelling (SD)	0.36				0.37			
Aedes albopictus + *Ae. aegypti*			12,654	12,694			12,668	12,708
Intercept	1.13	0.06			1.13	0.06		
Period								
Before	Ref.				Ref.			
Citywide	−1.54	0.09			−1.52	0.09		
Focal	−2.48	0.13			−2.47	0.18		
After	−0.07	0.11			−0.06	0.12		
Rainfall	0.15	0.04						
Temperature					−0.10	0.06		
Dwelling (SD)	0.28				0.29			
Aedes + *Culex* + *Limatus*			12,840	12,880			12,854	12,895
Intercept	1.21	0.05			1.20	0.06		
Period								
Before	Ref.				Ref.			
Citywide	−1.62	0.09			−1.60	0.09		
Focal	−2.54	0.13			−2.45	0.18		
After	−0.15	0.11			−0.11	0.12		
Rainfall	0.18	0.04						
Temperature					−0.14	0.06		
Dwelling (SD)	0.29				0.29			

SE, standard error; AIC, Akaike information criterion; BIC, Bayesian information criterion
