# Supplementary material for: Mosquito-Disseminated Insecticide for Citywide Vector Control and Its Potential to Block Arbovirus Epidemics: Entomological Observations and Modeling Results from Amazonian Brazil
Source: PLoS Med. 2017 Jan 17;14(1):e1002213. doi: 10.1371/journal.pmed.1002213 (PMC5240929; doi:10.1371/journal.pmed.1002213)
Supplement: S3 Table — Parameter estimates, standard errors, and values of the Akaike and Bayesian information criteria are provided. (PDF) [file pmed.1002213.s008.pdf]

S3 Table

S3 Table. Adult *Aedes* emergence: results of generalized linear mixed models with either rainfall or temperature as the weather covariate

Sex	Rainfall models				Temperature models			
	Estimate	SE	AIC	BIC	Estimate	SE	AIC	BIC
Females + males			11,226	11,266			11,235	11,275
Intercept	1.10	0.06			1.10	0.06		
Period								
Before	Ref.				Ref.			
Citywide	−3.23	0.10			−3.23	0.10		
Focal	−3.45	0.15			−3.43	0.19		
After	−0.19	0.10			−0.18	0.11		
Rainfall	0.12	0.04						
Temperature					−0.08	0.06		
Dwelling (SD)	0.37				0.37			
Females			9022	9062			9030	9070
Intercept	0.40	0.05			0.41	0.05		
Period								
Before	Ref.				Ref.			
Citywide	−3.13	0.11			−3.13	0.11		
Focal	−3.01	0.15			−3.01	0.19		
After	−0.25	0.10			−0.25	0.10		
Rainfall	0.11	0.04						
Temperature					−0.06	0.05		
Dwelling (SD)	0.30				0.30			
Males			8863	8903			8867	8908
Intercept	0.39	0.06			0.37	0.06		
Period								
Before	Ref.				Ref.			
Citywide	−3.20	0.12			−3.21	0.12		
Focal	−3.92	0.20			−3.81	0.23		
After	−0.11	0.10			−0.07	0.11		
Rainfall	0.11	0.04						
Temperature					−0.11	0.06		
Dwelling (SD)	0.39				0.40			

SE, standard error; AIC, Akaike information criterion; BIC, Bayesian information criterion
