# Supplementary material for: Mosquito-Disseminated Insecticide for Citywide Vector Control and Its Potential to Block Arbovirus Epidemics: Entomological Observations and Modeling Results from Amazonian Brazil
Source: PLoS Med. 2017 Jan 17;14(1):e1002213. doi: 10.1371/journal.pmed.1002213 (PMC5240929; doi:10.1371/journal.pmed.1002213)
Supplement: S1 Text — (PDF) [file pmed.1002213.s009.pdf]

**Supplementary Text S1**

## Original plan for statistical modeling

Our modeling strategy was relatively simple and was not prospectively written. Here we summarize our original plan.

We first emphasize that our focus was on *intervention effects*, which we initially planned to code as the four-period fixed factor we use in the main body of the paper. We also planned (i) to use a dwelling-ID random effect to account for repeated measures at the dwelling level, and (ii) to adjust for meteorological conditions (temperature and rainfall) and for varying sampling effort (via an offset specifying the (log)number of operational sentinel breeding sites [SBSs]). We anticipated that rainfall and temperature would likely be (negatively) correlated; we decided not to include both covariates in any single model if the correlation coefficient was >|0.60|.

Before seeing the data, however, we could not anticipate what error structure (Poisson or negative binomial) would best fit our mosquito count data (juveniles and emerging adults), so we planned to test this based on information criteria. Given our focus on population mean effects (i.e., fixed intervention effects across dwellings) rather than on dwelling-level variation (i.e., random effects), we planned to use AIC and BIC for this model evaluation; in doing this we follow Vaida S, Blanchard S. Conditional Akaike information for mixed-effects models. Biometrika. 2005;92: 351–370 (ref. [31] in the main body of the paper) and Greven S, Kneib T. On the behaviour of marginal and conditional AIC in linear mixed models. Biometrika. 2010;97: 773–789.

Finally, we thought that some operational problems might occur during PPF dissemination, so we kept track of any such problems through field observations. We however could not foresee what exactly those problems would be; the precise form of our alternative models testing the effects of different intervention coding schemes (lagged dissemination *vs.* dissemination intensity/quality) was therefore selected after seeing these data. As above, we planned to use AIC/BIC for these comparisons.
